# MYC in cancer: from undruggable target to clinical trials

**DOI:** 10.1038/s41573-025-01143-2

**Published:** 2025-02-19

**Authors:** Jonathan R. Whitfield, Laura Soucek

**Affiliations:** 1 Vall d’Hebron Institute of Oncology, Cellex Centre, Hospital University Vall d’Hebron Campus, Barcelona, Spain; 2 Institució Catalana de Recerca i Estudis Avançats, Barcelona, Spain; 3 Department of Biochemistry and Molecular Biology, Universitat Autonoma de Barcelona, Bellaterra, Spain; 4 Peptomyc S.L., Barcelona, Spain

## Abstract

MYC is among the most infamous oncogenes in cancer. A notable feature that distinguishes it from other common oncogenes is that its deregulation is not usually due to direct mutation, but rather to its relentless activation by other oncogenic lesions. These signalling pathways funnel through MYC to execute the transcriptional programs that eventually lead to the uncontrolled proliferation of cancer cells. Indeed, deregulated MYC activity may be linked to most – if not all – human cancers. Despite this unquestionable role of MYC in tumour development and maintenance, no MYC inhibitor has yet been approved for clinical use. The main reason is that MYC has long fallen into the category of ‘undruggable’ or ‘difficult-to-drug’ targets, mainly because of its intrinsically disordered structure that is not amenable to traditional drug development strategies. However, in recent years, attempts to develop MYC inhibitors have multiplied, and the first clinical trials have been testing their efficacy in patients. We are finally reaching the point at which its inhibition seems clinically viable. This Review will provide an overview of the different strategies to inhibit MYC, focusing on the most recently described inhibitors and those that have reached clinical trials.

## Introduction

*MYC* was recognised as an oncogene more than 4 decades ago and its importance in different aspects of tumorigenesis has only increased over time. It is a pleiotropic transcription factor that directs multiple intracellular and extracellular programs involved in tumorigenesis, encompassing essentially all hallmarks of cancers ^1–3^. Its activation by multiple upstream oncogenic signals makes it the perfect common target across different oncological indications and mutational profiles. However, for many years it was considered undruggable, as it did not fit into the standards established for traditional enzyme targeting and presented intrinsically disordered features not amenable to inhibition by small molecule inhibitors (SMIs) ^4^. MYC’s critical role in cell proliferation – and many other cellular processes – further enhanced its undruggable reputation, feeding the belief that side effects from its targeting would be highly deleterious in normal proliferating tissues.

Nevertheless, a variety of approaches have been taken to discover potential MYC inhibitors, and many molecules have shown *in vivo* efficacy against multiple cancer types in animal models ^3,5^. As a testament to the intense interest in this field, there are some 15000 hits in Pubmed for “MYC inhibition” in November 2024, with almost 1000 last year alone.

The development of Omomyc helped change the perception of MYC from undruggable, to a target that is ‘difficult-to-drug’ but worth further efforts. Omomyc is a 91 amino acid mini-protein that works as a MYC dominant negative and achieved a dramatic therapeutic impact in tumours, while causing only mild and reversible side effects in normal tissues ^6^. Recently, the first pharmacological tool derived from Omomyc (OMO-103) successfully completed a Phase I clinical trial, demonstrating safety and positive signs of drug activity. This now suggests that MYC could be finally declared ‘druggable’ and that a clinically viable MYC inhibitor is in sight ^7–9^.

In this Review we will provide a brief overview of MYC from its discovery to the current clinical trials, focusing on the most recent strategies that have reached clinical testing, with a special mention of our own OMO-103, but with an open mind towards alternative strategies that are quickly following and gaining momentum. Hopefully, these combined efforts will soon lead to the market approval of a MYC inhibitor that can benefit patients affected by multiple oncological indications.

## Fundamentals of MYC

**Figure 1:**
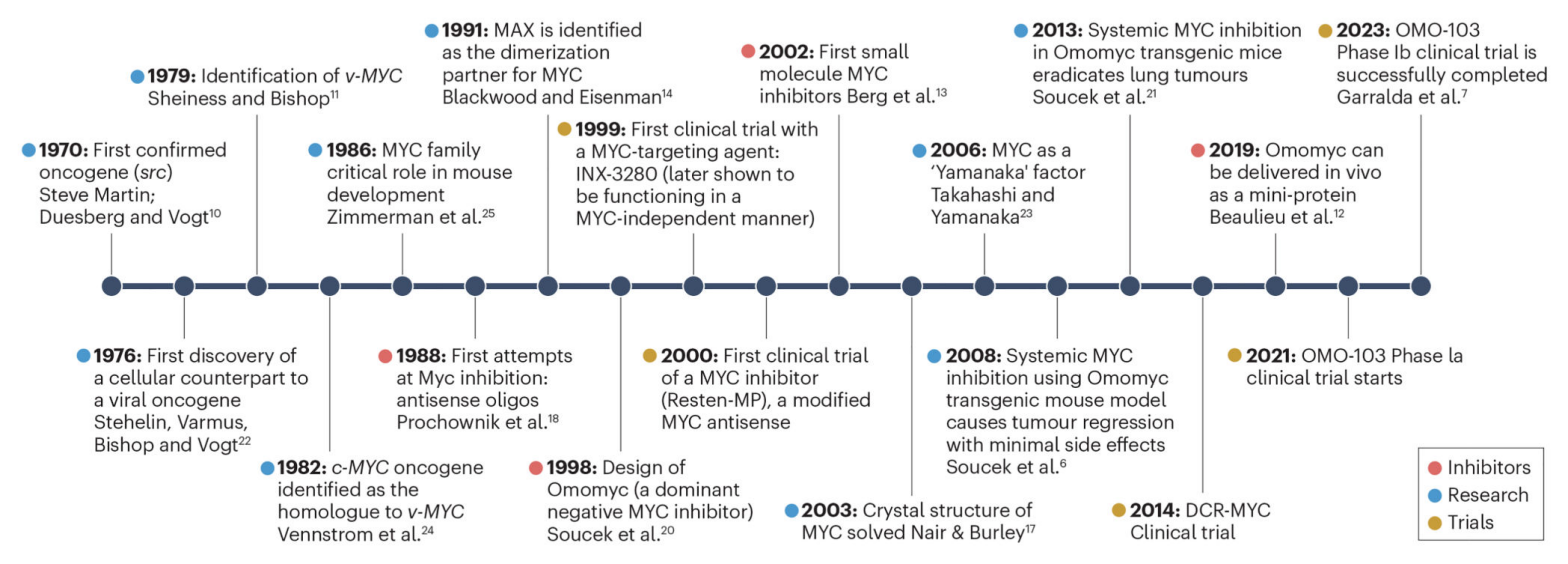
The timeline of MYC, from discovery to clinical trials **https://www.nature.com/articles/s41573-024-01060-w/figures/2**. This timeline starts with the discovery of the first oncogene in 1970 ^15,16^, and the identification of MYC itself in 1979 (v-MYC)^11^ and 1982 (c-MYC)^24^. An immense amount of research and number of publications then followed over the subsequent 4 decades, and a few key moments are indicated (shown with blue tags). Many strategies have been used to inhibit MYC, dating back to 1988^18^, and some milestones are indicated (shown with red tags). A number of inhibitors have progressed to clinical trials (shown with yellow tags), the first over 2 decades ago, while the first direct MYC inhibitor to successfully pass a Phase I trial occurred very recently (2023)^7^. This trial was based on Omomyc, a dominant negative MYC inhibitor designed back in 1998^20^ and used for many years to model MYC inhibition before its translation to the clinic^42^.

*MYC* was discovered some 40 years ago as the cellular homolog of a viral oncogene from an avian myelocytomatosis virus – the first retroviral oncogene found in the nucleus – called *v-myc*, which caused leukaemia and sarcoma in chickens ^10,11^. A timeline covering these decades from oncogene discovery to the first successful clinical trial is shown in Figure 1 ^6,7,11–25^.

The MYC family comprises 3 paralogs: the first identified was c-MYC, followed by MYCN (N-MYC; initially observed in neuroblastoma), and MYCL (L-MYC; identified in lung cancer) ^26,27^. They all belong to a network of transcription factors, called the Proximal MYC Network ^28^, that includes proteins sharing a similar DNA-binding and dimerization domain: the basic helix-loop-helix leucine zipper (bHLHZ). This network is centred around MYC’s obligate partner, MYC-associated factor X (MAX) ^14^, which is able to homodimerize, and to heterodimerize with MYC and with the functional antagonists MAX-dimerization proteins X (MXD1, MXD3, MXD4), MAX-binding protein MNT and MAX gene-associated protein (MGA) ^28^. Dimerization among the different members is determined by the HLHZ, whereas binding to DNA is dependent on their basic region ^29^. In this context, *MYC* transcriptional activity, both on activated and repressed genes, has been described to be strictly dependent on its binding to MAX ^30^.

Besides the bHLHZ domain at their C-terminus, MYC family members share an N-terminal transactivation domain (TAD) and a central region ^29^. These domains encompass highly conserved elements called MYC boxes, which interact with several co-factors involved in transcriptional control, but, in general, they lack a well-defined structure, limiting most of the structural studies for developing MYC inhibitory strategies to the bHLHZ domain ^4^.

MYC expression is normally tightly regulated and associated with transcriptional programs of efficient cell proliferation, such as tissue regeneration and wound healing. In physiological conditions, in fact, the MYC protein has a very short half-life of approximately 20 minutes ^31^, regulated through ordered phosphorylation, most notably of serine 62 and threonine 58, and proteasomal degradation after the dephosphorylation and ubiquitination of threonine 58 ^32^. However, MYC becomes deregulated in cancer as a result of gene translocation or amplification, or through its continuous activation and/or stabilization by upstream oncogenic signalling pathways such as Notch, Wnt/beta-catenin, Ras/PI3K/AKT/GSK-3 and Ras/Raf/ERK^1^. Tumour dependency on MYC is established through tonic signalling, rather than by MYC absolute levels^33^, and impinges on essentially all cancer hallmarks, from relentless growth, proliferation, protein synthesis, and altered cellular metabolism, to neo-angiogenesis and immune suppression ^3,34^.

All these features added MYC to the list of most pursued targets in cancer, prompting different research groups around the world to develop diverse strategies to inhibit it, encompassing direct and indirect approaches to counteract its function in cancer cells ^2,3,5^.

## Direct approaches to inhibit MYC

The very first MYC inhibitors entered clinical trials roughly two decades ago and, up until the recent trial of OMO-103, all had either been discontinued or not pursued further. Table 1 summarises the clinical status of direct MYC inhibitors ^7,12,35–44^. This section gives a historical perspective of the different approaches, with a particular focus on the most recent inhibitors, especially those that have reached clinical trials.

### Antisense and decoy oligonucleotides

**Figure 2:**
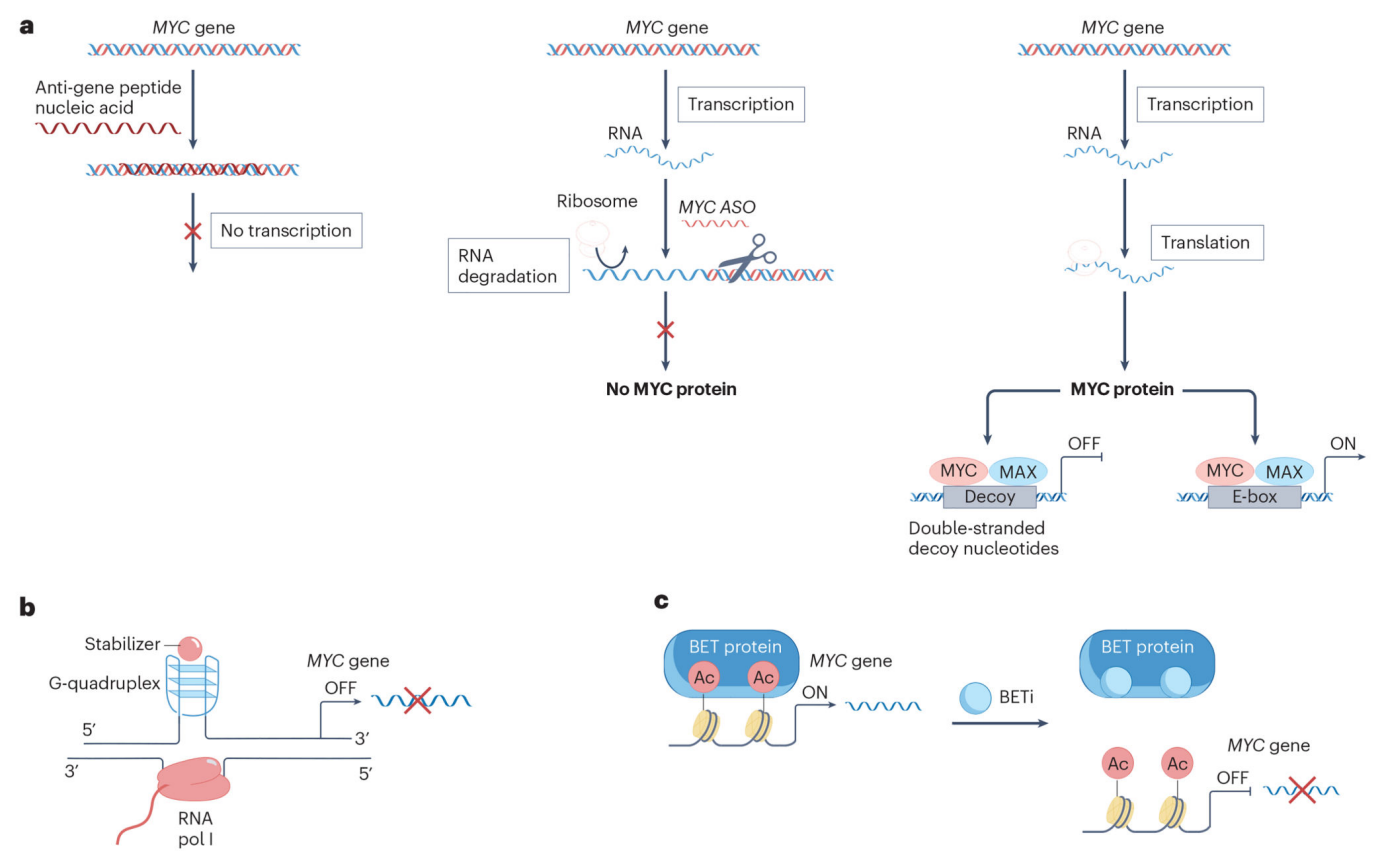
Inhibitors of *MYC* transcription and translation a. Oligonucleotide-based approaches to inhibit *MYC* can have different mechanisms of action. Anti-gene peptide nucleic acids (PNA) hybridize with complementary DNA within the *MYC* gene promoter thereby blocking its transcription; *MYC* antisense oligonucleotides (ASOs) interfere with *MYC* mRNA, blocking its translation, and double-strand decoy nucleotides compete with MYC targets for MYC/MAX binding, preventing their transcriptional activation. b. Binding of a stabilizer to the G-quadruplex prevents the advancement of RNA Pol I and transcription of the *MYC* gene. c. BET inhibitors (BETi) interact with BET proteins, preventing both their binding to the hyper-acetylated region of the *MYC* gene and the recruitment of the transcription machinery. This results in reduced *MYC* transcription.

The first approach to inhibit MYC was based on antisense oligonucleotides (ASOs) against *MYC* mRNA ^18,45^ (Figure 2A). Their initial success in murine erythroleukemia cells and in ras oncogene-transformed NIH 3T3 cells led to the clinical study by Inex Pharmaceutical of INX-3280, a chain of 15 nucleotide monomers (15-mer) with a phosphorothioate backbone originally developed by Arbutus Biopharma Corp. against the *c-MYC* oncogene. In preclinical regulatory studies in cynomolgus monkeys there were no clinically significant toxicities were observed upon intravenous injections ^43^. This reinforced the notion that cancer cells, but not normal cells, display exceptional sensitivity to MYC inhibition. Such cancer dependency on MYC is associated with the concept of ‘oncogene addiction’ [G], described over the years in multiple mouse models of cancer ^2,46^. INX-3280 reached phase I for the treatment of lymphoma and solid tumours in 2000 but was discontinued in 2002, after being repurposed as therapy against restenosis [G] ^47^, which was unfortunately not successful.

A modified form making use of a transmembrane carrier system, INXC-6295, reached clinical testing almost at the same time, but was abandoned owing to resource constraints. In any case, it was subsequently discovered that the anti-tumour effects of this ASO were likely due to immune stimulatory activity of a CpG motif within the molecule, rather than MYC inhibition *per se* ^48^.

Soon after, to improve *in vivo* stability and bioavailabilty of nucleic acids, AVI BioPharma (later renamed Sarepta Therapeutics) developed a phosphorodiamidate morpholino antisense oligomer (PMO) [G] called AVI-4126, which was shown to inhibit MYC expression in rats by preventing ribosomal assembly and mRNA translation ^49^. The compound reached clinical trials and showed bioavailability in patients with solid tumours ^36^. However, afterwards, it was mostly used in clinical trials in cardiovascular restenosis associated with neointimal hyperplasia [G], hence it was renamed RESTEN (Clinical Trial NCT00244647) and was not applied to oncology anymore.

Further improvement over the first PMOs has been achieved by lipid modification of the phosphorothioate backbone to increase drug delivery, cell permeability and stability ^50^. These modified compounds were used to target MYC in transgenic mouse models of MYC-driven primary hepatocellular carcinoma (HCC) and renal cell carcinoma (RCC), preventing tumour progression and eliciting intratumoral CD4^+^ T cell recruitment ^51^. Over the years, ASOs have been subject to different chemical modifications to enhance target affinity and stability. These include, for example, the locked nucleic acid (LNA), a conformationally restricted nucleotide containing a 2’-O,4’-C-methylene bridge, to lock the sugar into a C3’-endo conformation, increasing nuclease resistance and affinity for target mRNA ^52^, or a ‘gapmer’, a central gap region of 2’-deoxynucleotides flanked on both sides by modified nucleotides for the recruitment of RNase H and target mRNA cleavage ^53^. Recently, these modifications were used to design and synthesize a library of MYC-ASOs ^54^. These MYC-ASOs were able to decrease MYC mRNA and protein levels *in vitro* and *in vivo*, in a MYC-induced model of HCC, in which delivery of MYCASO every 3 days by tail vein injections reduced tumour burden and improved overall survival ^54^. It is important to note that, according to the authors, despite systemic administration, the therapeutic impact of MYCASOs in HCC might have benefited from their hepatotropism [G], which not only directed the therapy preferentially to the target tumour tissue, but also limited its distribution to other organs. This observation suggested that the selection of tissues that may be amenable to therapeutic oligonucleotides might be a wise choice for their future clinical application.

DNA-binding proteins like MYC can also be inhibited by double-stranded decoy oligodeoxynucleotides. These decoys carry consensus binding sequences – in *MYC’s* case the E-box – that ‘fish’ transcription factors, thus competing with endogenous DNA (Figure 2A). The use of decoy oligonucleotides against MYC goes back to the earlier days of MYC targeting. In 2005, a *MYC* decoy was coupled with the cell-penetrating peptide TP10 and a nuclear localization sequence (NLS), and this complex reduced the proliferative capacity of cancer cell lines ^55^. Later, a 20-mer double-stranded *MYC* transcription factor decoy was shown to decrease viability and modulate differentiation in a stem cell model ^56^. Development continues in this area with a recent report of more complex *MYC* decoy nanocomposites being synthesized that can inhibit growth of cancer cells ^57^. Although these decoy approaches are an interesting strategy, they have seemingly not been further pursued *in vivo*, likely dampening further enthusiasm for this approach and their development for clinical applications.

### siRNA and peptide nucleic acids (PNAs)

*MYC*-targeted oligonucleotide therapeutics have also been developed based on short/small hairpin RNA (shRNA) or small interfering RNA (siRNA). Historically, MYC was first successfully inhibited *in vitro* by lentiviral delivery of shRNA in normal and tumour human cell lines, interfering with cell cycle progression ^58^. A *MYC*-targeting siRNA called DCR-MYC delivered by lipid-based nanoparticles (LNPs), developed by Dicerna Pharmaceuticals, advanced to phase I trial (NCT02110563) in patients with advanced-stage solid tumours, multiple myeloma or lymphoma, and to phase Ib/II trials (NCT02314052) in patients with HCC. The liposomal delivery system was based on EnCore, Dicerna’s proprietary LNPs, characterized by a solid core of positively charged lipid, with an RNA payload inside. However, the studies were terminated on the sponsor’s decision, owing to a lack of sufficient gene silencing.

Recently, Hu *et al*. pre-compressed a *MYC*-targeting siRNA (sic-Myc) by octaarginine [G] and developed a lipoplex that was modified with a peptide derived from penetratin, called 89WP. This lipoplex was used to treat glioma-bearing mice by intranasal administration, and was preferentially internalized by glioma cells via active macropinocytosis, leading to downregulation of c-MYC mRNA and protein, resulting in death of glioma cells and prolonged survival of glioma-bearing mice ^59^. These results suggest that further improvement of siRNA encapsulation and tissue-specific delivery might eventually give this approach a second chance in clinical application.

Biogenera SpA is now leading the development of oligonucleotide therapeutics directed against undruggable proteins (including MYC and RAS) and non-coding RNAs ^60^. Their approach is based on specific gene expression inhibition at the level of DNA through an anti-gene peptide nucleic acid (agPNA) (Figure 2A). Their first compound, BGA002, is a *MYCN*-specific anti-gene oligonucleotide, which was designed against a unique sequence in the antisense DNA strand of exon 2 of *MYCN* and linked at its NH(2) terminus to a nuclear localization signal peptide ^61^. BGA002 was recently validated preclinically in combination with retinoic acid (RA) in a neuroblastoma cell line, where it reactivated neuron differentiation, while *in vivo* there was reduced tumour vascularization and significantly increased survival (50% survival was 33 days in the BGA002 treated group and only 21 days in the vehicle control group) in a *MYCN*-amplified neuroblastoma mouse model ^62^. In 2023, BGA002 was also shown to inhibit progression and increase survival in small cell lung cancer mouse models ^63^. The company is planning to initiate clinical trials based on these promising results. In the meantime, BGA002 obtained orphan drug designation for neuroblastoma from the European Medicine Agency (EMA) and from the Food and Drug Administration (FDA), and for soft tissue sarcoma from the EMA. The FDA also gave it “Rare Pediatric Designation” for neuroblastoma.

Other groups are pursuing a similar approach, with the recent publication of a gamma PNA conjugated to a nuclear localization signal showing efficacy in multiple xenograft models ^64^.

### G-quadruplex

Another means to interfere with *MYC* transcription is by leveraging the presence of a G-quadruplex (G4) structure in its P1 promoter (Figure 2B). G4 is a type of quadruple helix structure that originates from a continuous guanine-rich DNA sequence and can form under physiologically relevant conditions. Over 40% of human gene promoters contain at least one G4 motif, and as G4 formation is coupled to the establishment of accessible chromatin and nucleosome-depleted regions upstream of transcription start sites ^66^, genes marked by promoter G4s show higher expression levels compared to those without ^65^. The G4 displays different regulatory roles in biologically significant regions, such as human telomeres, promoter regions, replication initiation sites, and 5’- and 3’-untranslated region (UTR) of mRNA ^67^.^65,66^. In the case of the *MYC* promoter, this secondary structure is formed within the nuclease hypersensitivity element III 1 (NHE III 1) region, located upstream the P1 promoter ^68^. Since stabilized G4s can act as physical barriers preventing RNA polymerase from progressing along the DNA template ^69^, stabilizing G4 structures and consequently inhibiting the transcription of *MYC* and its expression has appeared as an opportunity not to miss to develop new cancer therapies and, in the past few years, a number of groups have contributed to the advancement of this field.

D089 is a benzofuran identified in a small molecule screen that stabilizes *MYC* G4 thanks to its planar aromatic structure, which allows it to stack on the external G-tetrads of the quadruplex with high affinity ^70^. D089 was effective against multiple myeloma cell lines, inducing G1 arrest and senescence, as well as multiple markers of endoplasmic reticulum stress and the unfolded protein response, as well as cell death by pyroptosis [G] ^71^.

Additionally, virtual screens have also been used to identify G4 stabilizers. One such screen identified a short peptide derived from the crystal structure of the bovine DHX36 helicase bound to *MYC* G4. It preferentially binds the *MYC* G4 with nM affinity ^72^. A combination of docking-based virtual screening and molecular mechanics/generalized Born surface area (MM/GBSA) free energy calculation on the FDA-Approved Drugs Library, led to the recent identification of trovafloxacin, ozanimod, and ozenoxacin as new *MYC* G4 stabilizers, suggesting their potential clinical use for MYC-related cancers ^73^.

Various derivatives have been designed and synthetized with the aim of developing G4 stabilizers. In 2023, a benzimidazolyl isoxazole derivative called EP12 was validated in primary multiple myeloma, both *in vitro* and *in vivo*, where it showed the ability to reduce MYC levels and disrupt the canonical nuclear factor-KB (NF-KB) signalling pathway ^74^. Also described in 2023, a synthesized benzoazole derivative called benzoselenazole m-Se3 was shown to stabilize the *MYC* G4, with growth inhibitory effects in hepatoma xenografts ^75^.

To improve G4 targeting, carbohydrates have been introduced into the structure, the rationale being that the sugar moiety can provide additional binding sites or interactions with the target protein, potentially improving compound selectivity. Coupling the imidazole derivative 19a with a D-glucose 1,2-orthoester, blocked *MYC* transcription and induced cell death in triple-negative breast cancer (TNBC) MDA-MB-231 cells, inhibiting tumour growth in a xenograft mouse model ^76^.

In addition, the design and synthesis of 7-aza-8,9-methylenedioxyindenoisoquinolines based on desirable substituents and π–π stacking interactions [G] were reported in 2024 as being able to stabilize *MYC* G4, while also inhibiting topoisomerase I ^77^. Their efficacy in targeting MYC was demonstrated *in vitro* in various cancer cell lines defined as MYC-dependent, and their therapeutic impact was shown *in vivo* in xenograft mouse models, including an orthotopic glioblastoma mouse model.

Others focused on *MYCN* and reported the discovery of a small molecule ligand that targets a pocket at the base of the hairpin region of the *MYCN* G4 with an affinity in the low μM range. An analogue of this compound, MY-8, was validated in NBEB neuroblastoma cells, where it slowed down cell proliferation ^78^.

In all the above studies, and those previously reported in the literature, the major concern is whether the anticancer activity of the compounds is really mediated by specific *MYC* targeting, since G4s are very similar throughout the genome. Some groups have therefore focused on improving MYC selectivity, for example by using a clamped DNA interference (DNAi) approach against the DNA structure in the *MYC* promoter ^79^. This has recently been extended to the development of a new and more selective molecule, DNAi 5T, which enters the nucleus, modulates cell viability, and decreases MYC expression in Raji B-cell lymphoma cells. The authors plan to develop this molecule through further preclinical models ^79^.

Another approach to improve G4 stabilizer selectivity for *MYC* is based on designing ligands that form multi-site interactions with flanking residues and loops of the G4 motif ^80^. With this strategy, downregulation of *MYC* and *hTERT* gene expression was achieved in MCF-7 cells, accompanied by senescence and DNA damage *in vitro*, and demonstrated anti-tumour activity in MCF-7 xenograft mouse models ^80^. According to the authors, the ligands were eliminated from the blood stream within 24 hours, suggesting that *in vivo* stability should be further improved.

On the other hand, in some cases, it was the lack of selectivity that revealed unexpected *MYC* G4 stabilizers. This happened, for example, in the case of APTO-253, which was initially developed to inhibit the mast/stem cell growth factor receptor gene *KIT* promoter, but that instead turned out to stabilize *MYC* G4, inhibiting MYC expression and inducing DNA damage in acute myeloid leukemia (AML) cells ^81^. APTO-253 was granted orphan drug designation for the treatment of AML by the US FDA and was tested in a phase Ia/b clinical trial (NCT02267863) in patients with relapsed or refractory AML (R/R AML) or high-risk myelodysplasias sponsored by Aptose Biosciences, Inc, starting in 2014. However, the study was placed on hold by the company in 2018, upon the report of an operational difficulty with an intravenous (i.v.) infusion pump at a clinical site, which initiated a thorough review of manufacturing and dosing procedures. Unfortunately, after 3 years on hold, the company announced the discontinuation of the program involving APTO-253.

### Small molecule inhibitors

**Figure 3:**
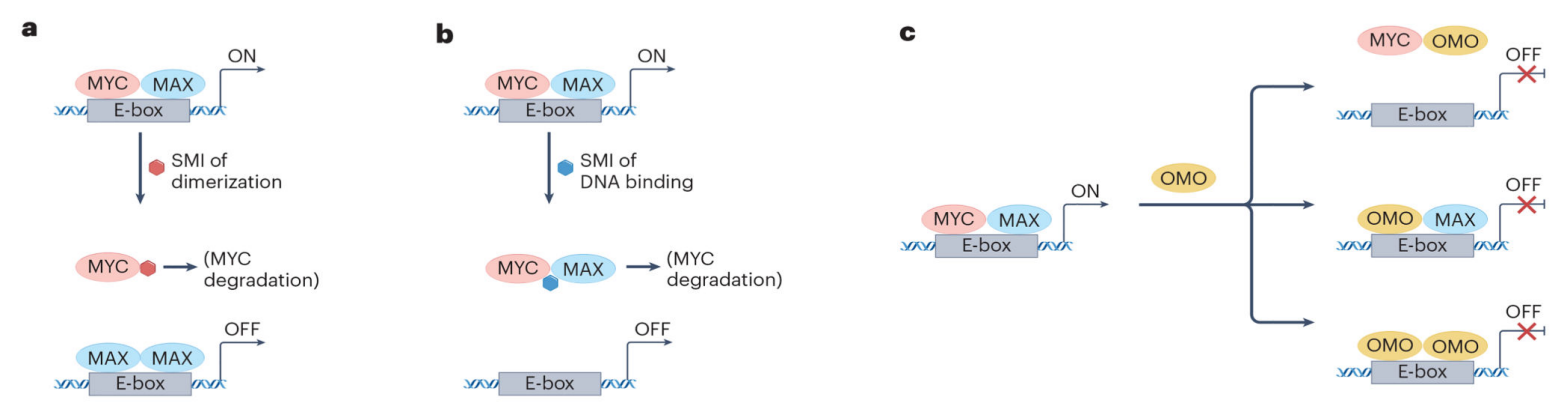
Inhibitors of MYC/MAX dimerization and DNA binding a. Small molecular inhibitors (SMIs) interfere with MYC/MAX dimerization. Once the dimer is destroyed, MYC is quickly targeted for degradation and MAX is free to homodimerize (or interact with other members of the MYC proximal network).. b. SMIs can also interfere with MYC/MAX binding to DNA, preventing the transcription of MYC target genes. c. Omomyc (OMO) acts through a triple mechanism of action whereby it sequesters MYC in MYC/OMO dimers unable to bind DNA, while also forming OMO/OMO and OMO/MAX dimers that occupy MYC target genes with inactive protein complexes. All 3 dimeric forms containing OMO are transcriptionally inactive.

To date, the most classic drug discovery approach used to attempt MYC inhibition remains the disruption of protein-protein interactions. However, as mentioned before, MYC lacks significant secondary and tertiary structure when not in complex with one of its biological partners, and therefore does not display the best features for SMIs ^29^. Most efforts have been directed to the disruption of MYC/MAX heterodimerization and binding to DNA (Figure 3A).

High-throughput screens have been the most common tool employed to identify MYC-targeting SMIs. For instance, in 2021, a GlaxoSmithKline small molecule library of 2 million compounds was screened using a Förster/fluorescence resonance energy transfer (FRET)-based assay — a technique used to assess molecular proximity— to detect MYC levels within cells and select compounds able to decrease them. This screen was followed up by triage assays to quickly eliminate toxic compounds, and qualify hits by fluorescence-activated cell sorting (FACS), cell growth assays and qPCR, generating three promising compounds that should be validated in future studies *in vivo* ^82^. It should be noted that, although the compounds affect MYC protein levels, data are not conclusive regarding whether they are direct MYC inhibitors and further studies are required for clarification.

To address the difficulty of identifying MYC regions suitable for targeting by SMIs, a cysteine-reactive covalent ligand screen was performed for compounds that could disrupt MYC binding to DNA and impair MYC transcriptional activity. They identified EN4, which covalently targets cysteine 171 of MYC, triggering downregulation of MYC target gene sets, as well as specific targets such as CDK2 and CDC25A, and impairing tumour growth in a mouse xenograft model of 231MFP breast cancer cells ^83^.

MYCMI-6, a SMI binding within the bHLHZ domain, was originally described in 2018 as being able to inhibit tumour cell growth in 60 human tumour cell lines and in a MYC-driven xenograft tumour model of neuroblastoma with IC50 concentrations as low as 0.5 μM ^84^. In 2021, the compound was also shown to prevent MYC interaction with MAX in breast cancer cell lines, inhibiting cell growth and inducing apoptosis with IC50 values 0.3 μM to >10 μM, with higher activity in the basal subtype ^85^. MYCi975, identified in 2019 in a *in silico* screening of a large chemical library combined with a rapid *in vivo* screen in mice ^86^, also blocks the MYC/MAX interaction, and has shown tolerability and efficacy *in vivo*, with reduced tumour growth in cell lines of MYC-dependent prostate cancer, Lewis lung carcinoma, and acute monocytic leukaemia cell lines in mouse allograft models ^86^. More recently, MYCi975 displayed IC50 values for growth inhibition from 2.49 to 7.73 µM in TNBC cell lines. Combined treatment with MYCi975 and either paclitaxel or doxorubicin resulted in more profound cell growth inhibition. Hence, the authors surmised that combination trials of MYCi975 should include either docetaxel or doxorubicin, and that MYC levels in tumour tissues could be used as a predictive biomarker, as they correlated with the response to the inhibitor in a panel of cell lines ^87^. MYCi975 also alters chromatin binding of MYC and the MYC network family proteins, and synergistically sensitizes resistant prostate cancer cells to enzalutamide and also oestrogen receptor (ER)-positive breast cancer cells to 4-hydroxytamoxifen ^88^. Synergy was also described in combination with a lysine-restricted diet to slow tumour growth in glioblastoma stem cells (GSCs) ^89^. In addition, MYCi975 inhibited head and neck squamous cell carcinoma growth in both a cell line derived xenograft and a syngeneic murine model inducing immune response through CD8+ T cell infiltration ^90^, underscoring a potential strategy to overcome cisplatin resistance ^91^. We are looking forward to the clinical development of this promising compound.

A ‘second-generation SMI’, 3JC48-3, decreased prostate cancer cell growth and viability *in vitro*, and showed tolerability and anti-tumour activity *in vivo* in a patient-derived xenograft (PDX) prostate cancer model when delivered intraperitoneally ^92^. Another SMI, B13 showed cytotoxicity activity against colorectal cancer (CRC) cells HT29 and HCT116 with IC50s of 0.29 μM and 0.64 μM, respectively, inhibiting binding of MYC/MAX dimers to DNA. B13 also inhibited HT29 growth in a xenograft mouse models at a dose of 40 mg/kg ^93^. In both cases, direct MYC inhibition has not been formally proven.

Potential SMI binding sites were identified through an *in silico* alanine scanning mutagenesis approach that revealed ‘hot-spot’ residues within the MYC/MAX interface. Tested compounds displayed non-covalent interactions with these hot-spot residues and were recently shown to interfere with MYC function and cancer cell growth *in vitro* ^94^. In this case, rigorous proof of target engagement of monomeric MYC was provided. *In vivo* experiments are eagerly awaited.

The SMI approach has also been applied to N-MYC, and MYCMI-7 can inhibit both MYC/MAX and N-MYC/MAX interactions in cells by inducing MYC and N-MYC degradation ^95^. In this study, sensitivity to MYCMI-7 correlated with MYC expression in a panel of 60 tumour cell lines and patient-derived primary glioblastoma and AML *ex vivo* cultures. Importantly, MYCMI-7 is also effective in mouse tumour models of MYC-driven AML, breast cancer, and *MYCN*-amplified neuroblastoma, where it inhibits tumour growth and prolongs survival ^95^.

Perhaps surprisingly, despite being the most common approach used so far and showing promising *in vivo* efficacy data in a number of models, SMIs have not yet undergone further clinical development with the exception of AntiMYCon (N77), reported as being under development for lung cancer, melanoma, and multiple myeloma, for which a phase I was planned in 2014, although there is no evidence that such a trial actually started to our knowledge.

### Peptide and mini-protein approaches

Interference with protein-protein interactions has also been the main objective in the use of peptides and mini-proteins against MYC.

The paradigm of this strategy has been established by our own Omomyc, a mini-protein of 91 aminoacids, validated as the most characterized MYC dominant negative to date. Omomyc was designed more than 2 decades ago, based on the bHLHZ sequence of human c-MYC, with 4 modified amino acids that change its dimerization capabilities ^20^. As such, whereas MYC can only dimerize with MAX, Omomyc forms dimers with MAX but also with itself, occupying the DNA with transcriptionally inactive complexes that interfere with E-box binding and transactivation. It also heterodimerizes with MYC, sequestering it in protein complexes unable to bind DNA ^20,96^ (Figure 3B). Several methods have been used to prove direct binding of Omomyc to MYC and MAX, including chimeric repressor dimerization assays — such as phage immunity tests and β-galactosidase or EMSA assays — published for the first time in 1998 ^20^, followed by co-immunoprecipitation (co-IP) experiments ^96–100^, fluorescence polarization assay, proximity ligation assay (PLA), double chromatin immunoprecipitation (ReCHIP) ^97^ and NMR ^12^.

Omomyc has been used extensively, by both our lab and other groups, mainly in its transgenic form, by viral infection of cancer cells with either inducible or constitutive expression cassettes, and by tissue-specific or switchable systemic expression in transgenic mice and xenograft mouse models (Box 1). These studies provided a proof-of-concept that MYC could be inhibited in multiple types of cancer and demonstrated reduced tumour cell proliferation, tumour regression and decreased metastasis ^42^. These models also served the purpose of establishing the feasibility and excellent therapeutic window of systemic MYC inhibition ^6,21,42,101^.

A key step to translating this proof-of-concept towards the clinic came with the discovery that the purified recombinant Omomyc protein itself possessed unexpected cell-penetrating properties, and could reach the nuclei and exert anti-MYC activity ^12^. This pointed towards the Omomyc mini-protein for the first time as a viable pharmacological entity, and a candidate for clinical development. As such, a formulated and intravenously-delivered Omomyc mini-protein, OMO-103, was developed by Peptomyc S.L. and entered first-in-human trials in 2021 in patients with all-comer advanced-stage solid tumour (NCT04808362). Omomyc showed safety, excellent pharmacokinetics (PK) and positive signs of target engagement and drug activity, with stabilization of the disease in approximately half of the 19 patients evaluated for response in the trial, and a 49% reduction of total tumour burden in a patient with metastatic pancreatic ductal adenocarcinoma (PDAC) ^7^. It is the first direct MYC inhibitor to have reached this milestone.

OMO-103 is now being tested in a Phase Ib study in combination with standard-of-care nab-paclitaxel– gemcitabine in first-line metastatic PDAC patients (NCT06059001) and in a Phase II study in advanced high-grade osteosarcoma patients (NCT06650514).

Other groups have worked to improve the Omomyc mini-protein delivery to cancer cells or its anti-MYC activity. One approach involved fusing Omomyc with phylomer delivery peptides (developed by Phylogica). The resulting FPPa-OmoMYC fusion was shown to induce apoptosis in TNBC cells *in vitro*, with efficacy in TNBC orthotopic allografts *in vivo* ^102^. Others approaches incorporated Omomyc into small protein scaffolds and nanocarriers ^103^ or, very recently, into a *Salmonella*-based delivery system, both as proof-of-principle of efficient cytosolic delivery of therapeutic molecules ^104^. In all cases, Omomyc is used as the benchmark for further development of anti-MYC protein therapeutics.

Helix 1 (H1) of the MYC HLH domain has also been used more than once as an inhibitor of MYC/MAX binding in preclinical studies. To aid its intracellular delivery, a fusion protein, Peptide Nuclear Delivery Device for H1 (PNDD1) was developed that links H1 to a non-toxic truncated form of *Pseudomonas* Exotocin A, which reaches the nucleoplasm via the endosome-to-nucleus trafficking pathway. This peptide inhibited MYC at nanomolar concentrations and is effective in multiple cancer cell lines ^105^. A thermally targeted version of H1, in which the H1 is coupled to both a cell-penetrating sequence and an Elastin-like polypeptide (ELP), was also used recently in glioblastoma rat models for thermal targeting ^106^, reducing tumour growth and increasing animal survival. These peptides have unfortunately not progressed to clinical testing yet.

A distinct peptide inhibitor has also started clinical trials, sponsored by IDP Discovery Pharma. The company began a phase I/II clinical trial with IDP-121, a short peptide that interferes with MYC/MAX binding and causes MYC degradation, for the treatment of refractory/relapsed hematologic malignancies (NCT05908409). Another stapled peptide, IDP-410, was designed by the same company to specifically target N-MYC. This peptide reduced glioma growth when administered systemically, even to orthotopic xenografts, demonstrating that it could cross the blood brain barrier (BBB) ^107^. There is no current information on whether this peptide will also proceed to clinical development.

### MYC degraders

**Figure 4:**
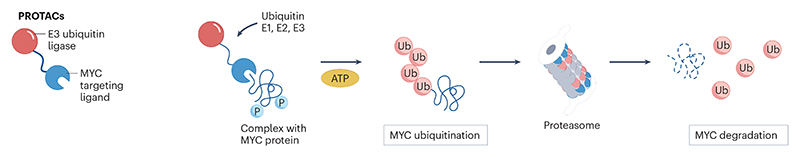
MYC degraders (e.g. PROTACS) Schematic representation of the mechanism of action of MYC degraders using PROTACS as representative example. PROTACS are heterobifunctional small molecule compounds consisting of a ligand for the target protein (MYC), a linker, and a ligand to recruit E3 ligase. Binding to MYC targets it for ubiquitination and degradation.

One of the most promising current technologies to target previously undruggable proteins uses proteolysis targeting chimaeras (PROTACS; Figure 4) or Cereblon E3 modulating drugs (CELMoDs), and more generally, protein degraders. However, in the case of MYC, this approach has been questioned because of the already short half-life of the protein, which tends to be continuously produced by cancer cells and might require frequent administration of any drug based on MYC degradation only. However, there are encouraging first results that might change this perception.

For example, a promising small molecule, WBC100, was shown to selectively degrade MYC and is effective in multiple mouse models after daily oral delivery ^44^. Co-IP and molecular docking experiments proved that this compound binds to the nuclear localization signal (NLS)1-basic-NLS2 region of MYC. Although other SMIs cause MYC degradation (such as MYCMI6, MYCMI7 or MYCi975), to our knowledge, this is the only one that has entered phase I clinical trials (in October 2021, sponsored by Zhejiang University) for MYC-positive tumours.

Recent PROTAC approaches include ProMyc, composed of a MYC targeting aptamer (identified in a high-throughput screen) as the ligand, coupled with the E3 ubiquitin ligase cereblon ^108^. ProMyc degrades MYC and after further modifications, could be delivered to treat a CRC mouse model by peri-tumoural injection. A threose nucleic acid aptamer conjugated to E-box DNA coupled with pomalidomide (a cereblon E3 ligase modulator) also showed MYC degradation. This conjugate, named TEP, had *in vivo* efficacy against a TNBC mouse model in combination with palbociclib ^109^.

A novel, alternate degrader approach was recently described in which an RNA-binding molecule is attached to a heterocycle that locally activates RNase L ^110^. A proof-of-concept was demonstrated with selective degraders for *MYC* mRNA that decreased MYC protein levels by 50%, caused cell cycle arrest, and reduced colony formation in cancer cell lines. It will be interesting to see how such degraders perform *in vivo*.

## Indirect approaches to inhibit MYC

Many more varied methods exist for targeting MYC indirectly, based on inhibitors of proteins other than MYC, that may be more readily targetable. Such targets may then regulate MYC transcription, translation, or stability, or even modulate alternative pathways that are required in MYC-driven tumours, creating the perfect condition for synthetic lethality (for specific reviews on the latter, please refer to^111,112^). There are advantages to these approaches as some inhibitors might already exist, but they may not be preferable owing to lack of specificity for MYC itself and potential off-target effects. These approaches are too numerous to be covered in detail here, but some are clearly worth mentioning for completeness.

One possible strategy is stabilization of the MAX homodimer, which then prevents the formation of MYC/MAX complexes. In fact, in 2019, a set of small molecule microarrays led to the identification of KI-MS2-008, an asymmetric polycyclic lactam, able to stabilize the MAX homodimer, causing consequent decrease of MYC protein and cancer cell growth *in vitro* and *in vivo* ^113^. Despite the promise held by this compound, we have not been able to find any further evidence of its development towards clinical application. Also, whether forced MAX homodimers can also affect the rest of the proximal MYC network remains to be established.

MAX sequestration in complexes that do not include MYC has also been attempted with the use of the first 146 amino acids of the MAX dimerization protein 1 (MXD1). This mini-protein was named Mad, and it displays cell-penetrating properties. To obtain Mad, the original sequence of MXD-1 was mutated at serine 145, which was replaced by an alanine to prevent its phosphorylation and rapid degradation. When used in cells, Mad was shown to bind to MAX and to E-boxes, blunting MYC binding to them, and curbing cell proliferation at concentrations lower than Omomyc ^114^. Unfortunately, no data are available regarding the use of Mad *in vivo* and no follow-up studies have been published to date.

A different rationale for indirect MYC inhibition is based on the notion that the *MYC* mRNA benefits from the presence of 5′ UTRs that favour its translation through engagement of the eukaryotic initiation factor 4F (eIF4F) translation complex. Targeting eIF4A1 — the helicase component of the eIF4F complex— by CRISPR/Cas9 or the SMI, silvestrol, could interfere with MYC translation, reducing proliferation and inducing death in experimental models of MYC-amplified G3-medulloblastoma ^115^. Preferential translation of *MYC* is also particularly abundant in chronic lymphocytic leukaemia (CLL), and synthetic flavagline FL3, a prohibitin (PHB)-binding drug, can inhibit eIF4F translation complex and prevent *MYC* translation in human and mouse CLL, causing growth arrest ^116^. The effect of this strategy is obviously not limited to *MYC* translation only. However, as eIF4F-targeting agents are in clinical trials, it would be interesting to see how they perform in MYC-high tumours.

The indirect strategy that has garnered much attention in recent years involves BET bromodomain inhibitors (BETi), epigenetically-directed, SMIs that target the bromodomain and extra terminal proteins, and showed promise to indirectly modulate MYC expression in cancer cells (such as JQ1, I-BET762, I-BET151 and OTX015) ^117^. BET proteins are known to directly activate oncogene transcription, including MYC itself, through recruitment to hyper-acetylated regulatory regions, where they favour the engagement of the core transcription machinery. Hence, inhibiting BET proteins results in reduced MYC expression (Figure 2C). Several BETi reached clinical trials in the past years. However, it has become clear that although MYC in some cases may be a major target of BETi, this is not the case in some tumours (such as osteosarcoma ^118^ or CRC ^119^), and these inhibitors can cause off-target toxicity, such as thrombocytopenia and pulmonary arterial hypertension ^120–122^. Moreover, in some instances, MYC expression levels seem unrelated to BETi efficacy ^118,123^. In fact, trial data have been unclear and have led to the conclusion that the approach needs further work to expand the biological knowledge of their complex mechanism of action in the context of MYC modulation ^124^.

The field of degraders has also been very active in the indirect inhibition of MYC, specifically related to the synthetic lethality context. Excitingly, MRT-2359 is a GSPT1 molecular glue degrader that entered a phase I/II trial in October 2022, sponsored by Monte Rosa Therapeutics (NCT05546268) in patients with selected MYC-driven solid tumours. GSPT1 is a translation termination factor that seems to be necessary for MYC-high tumour growth. Indeed, MYC-dependent tumours seem to require high rates of protein synthesis, and GSPT1 degradation decreases both protein translation and MYC transcriptional activity. Monte Rosa has announced interim PK/PD and clinical data in October 2023 indicating target engagement and encouraging signs of clinical activity in L-MYC/N-MYC high cancers.

Combining both approaches above, a BRD family PROTAC inhibitor was developed, called dBET1, that caused degradation of BRD2, BRD3 and BRD4, reducing MYC levels and showing efficacy in multiple AML cell lines ^125^ although no further development progress has been reported.

Epigenomic modulation has also been pursued by Omega Therapeutics, which recently started a phase I/II clinical study with their lead compound OTX-2002 in patients with HCC and other solid tumours associated with the *MYC* gene (NCT05497453). Although the specificity for *MYC* is not clear, this drug is based on a bicistronic mRNA coding for ZF-DNMT and ZF-KRAB proteins, delivered via LNPsFphamako and designed to downregulate *MYC* expression pre-transcriptionally, through epigenetic modulation ^19^. The company has recently announced preliminary data indicating encouraging safety, tolerability, and PK of the drug in a first pool of patients, raising hope and expectations for the upcoming ones.

Another proposed approach targeting MYC-dependent tumours is based on CDK9 inhibition. CDK9 is the kinase subunit of positive transcription elongation factor b (P-TEFb), that enables RNA polymerase II to transition from promoter-proximal pausing to productive elongation. CDK9 has been reported to control *MYC* transcription offering the opportunity to modulate its expression. In December 2022, Kronos Bio announced that their CDK9 inhibitor, KB-0742 reached target engagement and acceptable safety in the dose escalation phase of a phase I/II clinical trial (NCT04718675) and proceeded to phase II clinical trials in participants with relapsed or refractory solid tumours or non-Hodgkin lymphoma (NCT04718675).

### ‘Leftfield’ approaches

To find alternative strategies to target MYC, researchers followed also less orthodox paths. Some discovered, for example, that specific bacterial proteases can degrade MYC. Intravesical or peroral delivery of the recombinant bacterial protease Lon (rLon) decreased MYC levels and promoted survival in bladder and colon cancer mouse models suggesting that it could be used therapeutically ^126^. Interestingly, the authors also suggest that bacteria have thus evolved ways to control MYC levels in host tissue.

Another interesting finding came from the observation that the aureolic acid group antibiotics have anti-tumour activity. These antibiotics bind in GC-rich stretches in the DNA minor groove. One of the most well-known members of this category is mithramycin, used as a chemotherapeutic for several cancers and considered a MYC inhibitor (although non-specific) ^127^. Recent work shows that another member of the group – olivomycin A – inhibits transcription of *MYC* at nM concentrations ^128^.

Hyperthermia is another rather different approach that can reduce MYC levels and decrease tumour growth *in vitro* and *vivo*. Hyperthermia was induced in mouse models by immersing the subcutaneous tumours of anaesthetized mice in a 43°C water bath for 30 minutes ^129^. As with the antibiotic treatments, hyperthermia is unlikely to specifically affect MYC alone, but it is in current clinical practice as adjunct therapy, even if not commonly available.

Finally, attempts have also been made to use antibodies against MYC. Although antibodies have become commonplace in precision medicine, they are typically used for targeting proteins on the cell surface or in the cytoplasm, and not nuclear proteins. LA Cell was launched from Sorrento Therapeutics in 2015 to develop monoclonal antibodies against MYC. No further information is currently available. Very recently, an antibody-like molecule (an anti-MYC nanobody) was synthesized and shown to interact with MYC, and also internalize into cells, changing MYC-dependent gene expression and reducing proliferation ^130^.

## Conclusions & Perspectives

While writing this review, we realized with great satisfaction, that at this point it is surely a case of ‘when’ MYC will be a drugged clinical target, rather than ‘if’, which represents quite the paradigm shift over the last decade or so. The plethora of strategies for MYC targeting, both directly and indirectly, suggest that multiple options may eventually be available. At the same time, as we discussed previously ^5^, it was quite disappointing to notice that there are a number of inhibitor molecules that were promising, but were abandoned along the way, perhaps because of technical issues in their delivery or lack of funding. At least some of those could now merit revisiting in light of improved delivery methods, such as viral delivery of shRNA, and advanced techniques for nanocarrier encapsulation of SMIs or ASOs.

Even though clear biomarkers for ‘MYC addiction’ have yet to be defined, everybody agrees on the immense promise of a MYC inhibitor and its potential to be applied to multiple human cancers. This hopefully means that all the effort being invested into its targeting will end up with therapies that can benefit the highest possible number of patients. Furthermore, the roles of MYC in a wide range of cellular processes will likely extend the potential of its inhibition to the treatment of other diseases beyond oncology ^131^.

Another important consideration is that, owing to MYC’s pleiotropic effects and its role in resistance to treatments, combinatorial strategies with multiple current therapies should be considered and could be more than simply additive, producing synergistic results. Given the well-characterized role of MYC in immune-suppression ^132^, there is great promise for MYC inhibition in the context of immune oncology, for example, where it could significantly increase the success of immune checkpoint inhibitors ^133^. In addition, MYC overexpression seems to be a common feature of many therapy-resistant cancer cells, suggesting that such cancers may then be treatable – and in fact even more sensitive – to MYC inhibitors. Indeed, some groups have already proposed ‘evolutionary traps’ by which first line therapies could be used not only as a treatment, but also to induce selective pressure that will render the outgrowing resistant tumour acutely sensitive to second line treatments, like MYC inhibitors ^134^.

Albeit few, there are a number of trials assessing MYC-targeting compounds ongoing, which will hopefully provide further insights to move this field forward.

## Figures and Tables

**Table 1:** Past and current clinical trials with agents that directly target MYC.

Strategy	Mechanism	Example	Indication	Discontinued/ completed Successful Ongoing	References & clinical trial information
Direct inhibition of MYC expression	G-quadruplex stabilizers (prevent *MYC* transcription)	CX-3543 (Quarfloxin)	Neuroendocrine cancer	Discontinued	Phase II NCT00780663 Drygin et al. (2011)^37^, Brooks and Hurley (2010)^35^
		APTO-253	Relapsed/refractory acute myelogenous leukemia, myelodysplasia	Discontinued	Phase Ia/b NCT02267863
	Antisense oligonucleotides (prevent *MYC* translation)	INX-3280	Lymphoma and solid tumours	Discontinued	Phase I/II 1999–2002 Webb et al. (2001)^43^
		AVI-4126 (RESTEN-NG)	Cerebral Spinal Fluid of healthy volunteers	Discontinued	Phase I NCT00343148 Devi et al. (2005)^36^, Iversen et al. (2003)^38^, Kipshidze et al. (2003, 2004,2007)^39−41^
		RESTEN-MP	Coronary artery disease, coronary stent restenosis	Discontinued	Phase Ib, II NCT00244647 NCT00248066
	siRNA, microRNA (prevent *MYC* translation)	DCR-MYC	Solid tumors, multiple myeloma, or lymphoma, hepatocellular carcinoma	Discontinued	Phase I, Ib/II NCT02110563 NCT02314052
Interfere with MYC function	Mini-proteins or protein domains (interfere with MYC function)	OMO-103 (Omomyc)	All-comers solid tumours	Successful	Phase I/IIa NCT04808362 Beaulieu et al. (2019) ^12^, Massó-Valles et al. (2021)^42^ Garralda et al. (2024)^7^
			metastatic PDAC	Ongoing (2026)	PhaseIb NCT06059001
			Advanced High-grade Osteosarcoma	Ongoing (2026)	Phase II NCT06650514
		IDP-121	Relapsed/refractory hematologic malignancies	Ongoing (2026)	Phase I/II NCT05908409
Epigenetic modulators	mRNA therapeutic causing epigenetic changes	OTX-2002	Hepatocellular carcinoma and other solid tumours known for association with MYC	Ongoing (unknown)	Phase I/II NCT05497453
Degrader	Targets NLS region of MYC and induces its degradation	WBC100	MYC-positive advanced solid tumors	Ongoing (unknown)	Phase I NCT05100251 Xu et al. ^44^(2022)

NCT clinical trial numbers are indicated, when available, with estimated study completion dates for ongoing trials shown in parentheses.

## Box 1 Transgenic Omomyc: a versatile MYC dominant negative before its development into a pharmacological tool

Before the discovery of the cell-penetrating activity of the purified Omomyc mini-protein, this research tool was mainly used as MYC dominant negative by transgenic expression using various approaches:

In vitro models: In this context, the *Omomyc* transgene was expressed through viral infection of cancer cells with inducible or constitutive expression cassettes. In the case of inducible expression, both doxycycline- and 4-hydroxytamoxifen-dependent constructs were employed^20,42,96,98,100,135–139^.

In vivo models: In this case, the *Omomyc* transgene was cloned under the control of tissue-specific or systemic promoters, used then to either generate transgenic mice or to infect commercial or patient-derived cells, which were then transplanted into mice. Once again, its expression was either constitutive or switchable by addition of doxycycline to the drinking water or by tamoxifen administration to the animals^6,21,42,98,101,135,136,138–142^.

Key findings from these studies:

- Proof-of-concept for MYC inhibition and its consequences in multiple cancer types
- Consistently reduced tumour cell proliferation and increased apoptosis across different oncological indications
- in vivo tumour regression and/or eradication
- Decreased metastasis

Significance:

These models established the feasibility, safety and excellent therapeutic window of systemic MYC inhibition, prompting further development of Omomyc into a pharmacological tool.

## Glossary

Oncogene addiction: phenomenon in which cancer cells become heavily dependent on one or a few specific genes for their growth and survival
Restenosis: the recurrence of blood vessel narrowing upon angioplasty and stenting
Phosphorodiamidate morpholino antisense oligomer (PMO): short single-stranded DNA analogs that are built upon a backbone of morpholine rings connected by phosphorodiamidate linkages
Neointimal hyperplasia: post-intervention, pathological, vascular remodelling due to the proliferation and migration of vascular smooth muscle cells in the tunica intima layer, causing vascular wall thickening and gradual loss of luminal patency
Hepatotropism: preferential homing to the liver
Octaarginine: a sequence of 8 arginines (R8)
Pyroptosis: an inflammatory form of lytic programmed cell death that occurs most frequently upon infection with intracellular pathogens as part of the antimicrobial response
π-π stacking interactions: π-π stacking interactions are noncovalent attractive forces that occur between aromatic rings or other π systems
